# Acknowledgement to Reviewers of *Marine Drugs* in 2015

**DOI:** 10.3390/md14010025

**Published:** 2016-01-21

**Authors:** Marine Drugs Editorial Office

**Affiliations:** MDPI AG, Klybeckstrasse 64, CH-4057 Basel, Switzerland; marinedrugs@mdpi.com

The editors of *Marine Drugs* would like to express their sincere gratitude to the following reviewers for assessing manuscripts in 2015. 

We greatly appreciate the contribution of expert reviewers, which is crucial to the journal’s editorial decision-making process. Several steps have been taken in 2015 to thank and acknowledge reviewers. Good, timely reviews are rewarded with a discount off their next MDPI publication. By creating an account on the submission system, reviewers can access details of their past reviews, see the comments of other reviewers, and download a letter of acknowledgement for their records. In addition, MDPI has launched a collaboration with Publons, a website that seeks to publicly acknowledge reviewers on a per journal basis. This is all done, of course, within the constraints of reviewer confidentiality. Feedback from reviewers shows that most see their task as a voluntary and mostly unseen work in service to the scientific community. We are grateful to our reviewers for the contribution they make.
Abbott, WadeHildebrand, MarkPark, Ro-DongAbdel-Wahhab, Mosaad A.Hillig, FriederikePark, Pyo-JamAbe, KatsuyaHoffman, Angela M.Park, YoonkyungAbraham, Wolf-RainerHolloway, Graham P.Park, Cheol WheeAbraham, AnnHosaka, TakeshiParolini, CinziaAbreu, PedroHosokawa, MasashiPatel, AtishAfiyatullov, Shamil Sh.Hosokawa, SeijiroPattenden, GeraldAfonso, Clélia N.Hosono, MasahiroPavon-Djavid, GracielaAftimos, PhilippeHossain, ZakirPenton, C. RyanAgarwal, AnupriyaHsu, Hsien-YehPereira, DavidAgeitos, Jose ManuelHsu, Day-ShinPereira, LeonelAgyei, DominicHsu, Jue-LiangPereira, FlorbelaAki, TsunehiroHu, X. T.Pereira-Wilson, CristinaAlbalat, RicardHuang, YanPerkins, Laura E. L.Aleixo, Inês MarquesHuang, Chun-YinPertusati, FabrizioAllais, FlorentHuang, LiPerumal, DeepakAllard, Pierre-MarieHuang, Tse-HungPetek, SylvainAltieri, BarbaraHuang, Yu-TzuPetrakis, Panos. V.Altmann, FriedrichHubert, JanePetzelbauer, PeterÁlvarez, MercedesHucho, FerdinandPicot, L.Amat, MercedesHughes, HelenPiggott, AndrewAminin, Dmitry L.Huigens III, Robert W.Pinchetti, Juan Luis GómezAnastyuk, Stanislav D.Humpage, AndrewPires, José Carlos MagalhãesAndjelkovic, Anuska V.Hwang, Pai-AnPistone, AlessandroAndrade, PaulaHyun, Bae KiPleissner, DanielAndrianasolo, EricChung, Hyun-JeongPlieger, PaulAnesi, AndreaIanora, AdriannaPlourde, MélanieAnnaert, WimIgarashi, YasuhiroPochet, RolandAnraku, MakotoIkarashi, YoshiakiPollegioni, LoredanoApostolidis, EmmanouilIlies, Marc A.Polyak, StevenArahuetes, Rosa MªImhoff, Johannes F.Pombo, AnaArai, MasayoshiImokawa, GenjiPomin, Vitor H.Aranaz, InmaculadaImperatore, ConcettaPotin, PhilippeArand, MichaelInagaki, KenjiPrado, SoizicAranda-Rodriguez, RocioInceoglu, BoraProteau, PhilAraoz, RómuloIoannou, EfstathiaPrzytulska, AnnaAriga, KatsuhikoIrrgang, K.-D.Puddick, JonathanArizza, VincenzoIsaza Martínez, José HipólitoPurcell, Erin K.Arthur, GilbertIshmael, Jane E.Radke, Elizabeth G.Attoub, SamirIssinger, Olaf-GeorgRafael, DianaAustin, Rachel N.Itoi, S.Ragavendra, H. BalajiAvato, PinarosaIvanova, Elena P.Raj, Madhwa H.G.Azam, LaylaIyer, ArunRamadan, UsamaAzerad, RobertJackson, PhilippaRao, Kosagi SharafAzzi, GeorgeJacobsen, JetteRasenick, Mark M.Bagno, AlessandroJacobso, Peer B.Rateb, Mostafa E.Baker, Bill J.Jang, JaehyukRathinavelu, AppuBakovic, MaricaJennings, PaulRawlings, Neil D.Ballatore, CarloJensen, Liselotte E.Rebek, JuliusBalunas, MarcyJenssen, HåvardReen, JerryBanack, SandraJeon, You-JinReguera, BeatriceBanskota, Arjun H.Jesus Raposo, Maria FilomenaReitzel, AdamBaptista, Mafalda S.Jiao, GuanglingReyes, FernandoBarba, Francisco J.JIménez, CarlosReynisson, JóhannesBarbosa, Judite N.Jiménez-Barbero, JesúsRho, Jung-RaeBarbosa, André L. R.Jin, ZhendongRiccio, RaffaeleBaron, RégisJoo, Hong-GuRiccioni, GrazianoBarros, MarceloJoshi, LokeshRiedel, Richard F.Bartual, AnaJoullié, Madeleine M.Rimbach, GeraldBates, StephanJun, Joon-YoungRivera, GustavoBeach, DanielJung, JeeRizzo, ManfrediBeaulieu, LucieJung, Woo-JinRizzo, Angela MariaBelas, RobertJung, Won-KyoRocha-Santos, Teresa A.Bendik, IgorJustino de Almeida, CelineRodrigues, DinaBenkendorff, KirstenKaas, QuentinRodríguez, JaimeBerdyshev, EvgenyKalinin, VladimirRoh, ChanghyunBernstein, JonathanKamat, Pradip K.Rokstad, Anne MariBerrue, FabriceKamata, TamihiroRolland, J.L.Berry, JohnKamdem, Ramsay S. T.Romano, GiovannaBetancor, M.B.Kanakkanthara, ArunRosa-Molinar, EduardoBhattacharjee, MeenakshiKaruso, PeterRosic, NedeljkaBianco, I.D.Kashino, YasuhiroRoss, Ian LBianco, RobertoKashiwagi, SatoshiRoullier, CatherineBickmeyer, UlfKashman, YoelRousseau, G.Bifulco, GiuseppeKasiotis, Konstantinos M.Roussis, VassiliosBlanco, JuanKaszowska, MartaRoze, Ludmila V.Blomme, EricKatikou, PanagiotaRufini, StefanoBlunt, JohnKaufmann, KatrinRuiu, LucaBodmer, DanielKem, William R.Rupaimoole, RajeshaBogel-Łukasil, RafałKerch, GarryRupasinghe, Thusitha W.T.Bonjoch, JosepKerr, RussellRupérez, PilarBorea, Pier AndreaKhadka, ManojRussell, Fraser D.Botana, LuisKhanna, RajeshRusso, Gian LuigiBouaïcha, NoureddineKhanna, AnchitRustad, TuridBouteau, FrançoisKhiari, ZiedRustenbeck, IngoBowden, Bruce F.Khora, Samanta S.Rzymski, PiotrBradley, Walter G.Khurana, SurenderSaha, Sushanta KumarBrand, KorbinianKigoshi, HideoSaito, NaokiBreitbart, HaimKijjoa, AnakeSaito, AkihikoBrinchmann, Monica F.Kikuchi, JunSakai, RyuichiBrock, MatthiasKim, Jung BaeSakamoto, ToshioBrown, DarenKim, Hye SunSala, Gerardo DellaBrzezinski, RyszardKim, Sang MooSaludes, Jonel P.Budroni, MarilenaKim, Hyeung-RakSamuelsen, Anne Berit C.Bugni, TimKim, YoungsooSanches-Silva, A.Bulone, VicentKim, Hyung SikSansone, ClementinaBurrows, HughKingston, David G. I.Santander, JavierBurton, Edward A.Kinnunen, ViljamiSantini, AntonelloButler, Mark S.Kittakoop, PrasatSantos, ValentínCabado, Ana G.Kiuru, PaulaSantos, Sónia A. O.Cabral, JaydeeKlettner, AlexaSantos, AbelCaffrey, PatrickKlueter, AnkeSantos Costa de Morais, Rui ManuelCai, LishengKluza, JéromeSarabia, FranciscoCai, JingminKnobloch, Juergensarigiannis, YiannisCalarco, AnnaKnölker, Hans-JoachimSarojini, VijayalekshmiCaldwell, Kim A.Kobayashi, ToshihideSass, HenrikCallery, Patrick S.Kobayashi, HidekiSasso, SeverinCalvanese, VincenzoKolaczkowski, MarcinSatake, MasayukiCamp, Jason E.Koller, MartinSato, ShigeruCampbell, ChristopherKomatsu, MasaharuSatomi, YoshikoCañavate, JoséKong, Chang-SukSatomi, MasatakaCapel, FrédéricKong, Siu-KaiSavage, Thomas J.Capon, RobertKonno, HiroyukiSayers, Thomas J.Caramujo, Maria JoséKonoki, KeiichiScaife, Mark A.Carballeira, Nestor M.Korsnes, Mónica SuárezSchaberle, Till F.Carbone, AnnaKozlov, Sergey A.Schauss, Alexander G.Carmeli, ShmuelKrause, MirjaSchiraldi, ChiariCasettari, LucaKrause, Jeffrey W.Schmalz, Hans-GuentherCastell, AlinaKrock, BerndSchmelzer, Christian E. H.Castellano, FlaviaKubo, TaiSchnermann, Martin J.Castle, Neil A.Kulminskaya, Anna A.Scholz, BettinaCautain, BastienKumar, VijaySchröder, Heinz C.Cenac, NicolasKuo, Jen-MinSchroeckh, VolkerChai, Christina L.L.Kurra, YadagiriSchweikart, KarenChakrabarti, DebopamKusaykin, MikhailScognamiglio, VivianaChand, SubhashKushmaro, ArielSeca, Ana M. L.Chang, Hsueh-WeiKuyucak, SerdarSégui, BrunoChang, GeoffreyKwak, Jong-YoungSeipke, RyanChang, Fang-RongKwon, Hyung-JinSelwood, Andrew I.Chang, Chi-IKyzas, George Z.Seo, Jung-KilChang, YaoguangLa Motta, ConcettinaSeo, JungChao, Louis KuopingLaBarbera, Daniel V.Sepe, ValentinaChauvierre, CédricLabes, AntjeSerra, StefanoChen, Qi-YinLaborie, Marie-PierreSethi, GautamChen, RongLadrat, Christine DelbarreSeubert, Erica L.Chen, Jiann-ChuLafleur, JohnShikov, Alexander N.Cheng, Yuan-BinLai, DaowanShim, Sang HeeCheng, Kuan-ChenLangenbach, DorotheeShimizu, ToshiyukiCheung, Randy Chi FaiLanzetta, RosaShin, JongheonChevrot, RomainLatortue, Marie C.Shin, Hyeon-CheolChiang, Meng-TsanLavoie, Jean-PierreShindo, KazutoshiCho, YukoLawrence, William S.Shiozaki, KazuhiroChoi, Jong-IlLay, Jackson O.Shoguchi, EiichiChoi, HyukjaeLe Hegarat, LudovicSiah, AhmedChoi, Yung HyunLeão, Pedro N.Siddiqui, Khawar SohailChoi, Eun-MiLeaver, Michael J.Silva, Tiago H.Choi, Young HaeLee, Sang KookSilva, LuísChoi, YoungLee, Yeon-JuSimonini, RobertoChoshi, TominariLee, VictorSingh, ManishChristaki, EfterpiLee, SeokjoonSiodłak, DawidChristos, GeorgiouLee, Ok-HwanSipos, PálChu, JustinLee, Jun SikSirangelo, IvanaChun, Byung-SooLee, Jae-HoSison-Mangus, MarilouCiancia, MarinaLee, Hyung HoŠkalko-Basnet, NatašaCiardelli, GianlucaLee, KyoungSmith, GordonCicenas, JonasLeeper, Finian J.Smith, Thomas J.Cicero, Arrigo F.G.Leistner, EckhardSmith, AlanCipres, Miriam SolanaLeone, AntonellaSmith, Kirsty F.Cirrincione, GirolamoLi, JieSmith, WanliClark, RichardLi, PengSmith, G. JasonCocquerel, LaurenceLi, JunSmith-Palmer, TruisCoelhoso, Isabel M.Li, YanSolaiman, Daniel K.Y.Colliec-Jouault, SylviaLiefer, Justin D.Solo-Gabriele, HelenaCollins, MauriceLiekens, SandraSolovchenko, AlexeyColonna, GiovaniiLiew, Lydia P.P.Sosa, SilvioConcas, AlessandroLim, Sang-BinSpanò, AntonioCondezo-Hoyos, L.Lima, Marcelo A.Speck, Peter G.Conery, AnnieLin, ZhenjianSperanza, LorenzaCorazzi, LanfrancoLisa, María NataliaSpoof, LisaCorsaro, MariaLittle, R. DanSprague, M.Costa, Pedro ReisLittlefield, Bruce A.Stancheva, RosalinaCosta, Rosaria M. R.Liu, HongbingStange, ClaudiaCosta-Lotufo, Leticia VerasLiu, DehuaSteger, GerhardCosta-Rodrigues, JoãoLiu, ZhanzhuStevenson, Roselynn M.W.Costantini, MariaLiu, JinStoskopf, MichaelCostantino, ValeriaLiu, JunyingStournaras, ChristosCouderc, FrançoisLiu, HaijunStrongin, Robert M.Cozza, GiorgioLlewellyn, Carole A.Su, Wen-DaCruz-Rivera, EdwinLobo Castañón, Maria JesusSu, ZhiCzjzek, M.López Contreras, AnaSu, Jui-HsinD’abrosca, BrigidaLouzao, M. CarmenSucunza, DavidD’argenio, GiuseppeLovely, Carl J.Sumi, ShoichiroD’Auria, ValeriaLowenthal, Ray M.Sung, Ping-JyunDaboussi, FayzaLu, JunSung, Mi JeongDahms, Tanya E. S.Lu, Mei-ChinSupuran, Claudiu T.Daly, NorelleLüdeke, SteffenSurup, FrankDaranas, Antonio HernándezLuesch, HendrikSwanson, Geoffrey T.Dasgupta, SayaniLuna, Gian M.Tachibana, ShinjiroDaubenspeck, JamesMa, Dik-LungTaghibiglou, ChangizDay, John G.Ma, DongyingTaglialatela-Scafati, OrazioDe Agostini, ArianeMacCarthy, EugeneTakada, KentaroDe Geest, BartMacedo, AlexandreTakahashi, KoretaroDe La Cruz, Armah A.MacMillan, JohnTakahashi, DaisukeDe Los Ríos, Cristóbal Macrander, JasonTakano, KatsuraDe Marco, ArioMadhukar, BurraTanaka, TsuyoshiDe Pascale, DonatellaMaeda, HayatoTanaka, TakujiDe Pauw, EdwinMaehre, Hanne K.Tanner, Julian AlexanderDebelle, LaurentMaggs, ChristineTantillo, DeanDelfourne, EvelyneMancini, InesTarman, KustiariyahDenny, BillMangoni, AlfonsoTartaglione, LucianaDeRosa, MariaMani, SridharTava, AldoDi Pietro, AttilioManickam, KrishnanTeas, JaneDiamond, GillMarchetti, PhilippeTedeschi, GabriellaDiana, PatriziaMarechal, EricTeixeira, NatérciaDias, Daniel A.Mariadhas, Valan ArasuTheodosiou, EleniDíez, DavidMarie, BenjaminThomas, OlivierDittmann, ElkeMarigomez, IonanTiritan, Maria ElizabethDiz, Angel P.Marino, FrancaTomás, JuanDoi, TakayukiMariottini, GianluigiTommonaro, GiuseppinaDolashka, PavlinaMartel, FátimaTomoda, HiroshiDominguez, HerminiaMartin, Luc J.Torzewski, MichaelDoyle, TomMartin-Portugues, G.Tosco, AlessandraDuca, LaurentMartinez-del-Pozo, AlvaroToubiana, MyleneDuda, KatarzynaMartyniuk, Christopher J.Trepel, MartinDuncan, KatherineMatsumi, RieTresini, MariaDwivedi, ChandradharMaupin, Owen M.Trincone, AntonioDyshlovoy, SergeyMcCall, Jennifer R.Trougakos, IoannisEberhart, Bich-Thuy L.McCarron, PearseTsai, Yow-FuEckhart, LeopoldMcCarthy, FlorenceTsuda, MasashiEdrada-Ebel, RuAngelieMcCarthy, PeterTsurkan, Mikhail V.Eilertsen, Karl-ErikMcCarty, MarkTsutsui, ShigeyukiEirín-López, José M.Mcdougall, Gordon J.Tubaro, AureliaEkker, MarcMcGenity, TerryTulla-Puche, JuditElkins, Jonathan M.McGlory, ChrisTurner, AndrewElofsson, MikaelMcMeans, Bailey C.Twelves, ChristopherEsteban, Maria AngelesMcMullin, David R.Tzartos, SocratesEstepa, AmparoMcPhail, KerryUsami, YoshihideEsteves, Ana I. S.Mebs, DietrichVajdovich, PeterEvans, FranklinMechler, AdamVale, PauloEvans, Bradley S.Medina, Miguel AngelValentao, PatríciaEzquerra-Brauer, Josafat-MarinaMeloni, BrunoValverde, Ángela M.Fabris, MicheleMendiola, Jose A.Van Altena, IanFaggio, CaterinaMeng, YizhiVan Berkel, WillemFairlamb, IanMeng, MenghsiaoVan Cutsem, PierreFalconer, Ian R.Menna, MarialuisaVan Den Broek, A.E.M.Falque, E.Meriluoto, JussiVan Doren, Steven R.Fang, Evandro FeiMészáros, TamásVan Griensven, Leo J.L.DFardin-Kia, Ali Reza K.Metsä-Ketelä, MikkoVan Lanen, StevenFareed, JawedMeyer, AnneVanhaecke, LynnFaria, Tiago Q.Mi, Fwu-LongVarela, JoãoFauchot, JulietteMiccadei, StefaniaVarese, CristinaFaulkner, SimonMichaud, PhilippeVårum, Kjell M.Fenice, MassimilianoMichiels, JorisVasconcelos, VitorFernández, José J.Milledge, John J.Vasiljevic, TodorFerrando, SaraMiller, JohnVázquez, JulioFessard, ValerieMiller, Aaron W.Vejux, AnneFewer, DavidMineva, TzonkaVellecco, ValentinaField, RobertMing, Li-JuneVenkatesan, JayachandranField, JessicaMinond, DmitriyVenkateswaran, KasthuriFigueroa, Félix LópezMisevic, Gradimir N.Vergara, DanielFitton, HelenMishra, SandhyaVesterkvist, PiaFlaim, GiovannaMiyashita, KazuoVetter, WalterFleury, YannickMohanam, SanjeevaVetvicka, VaclavFontana, AngeloMolgo, JordiVidanarachchi, Janak K.Forino, MartinoMolinski, TadeuszVik, AndersFranco, ChrisMölleken, HelgaVílchez, CarlosFrank, SašaMonteiro, Robson Q.Villaverde, Juan JoséFrankenberg Dinkel, NicoleMonti, Maria ChiaraVinogradov, EvgueniiFranzellitti, SilviaMoon, Il SooVinogradov, EvgenyFreitas, Ana C.Moratti, S.C.Viola, GiampietroFreund, NadjaMorel, FrançoisVon Lintig, JohannesFriedman, Melissa A.Morikawa, HisaoVougogiannopoulou, KonstantinaFrisvad, Jens ChristianMorioka, TomoakiWaite, J. HerbertFujii, MikioMorita, NaokiWakimoto, ToshiyukiFuwa, HaruhikoMorris, Gordon AWan, MinGall, BrianMorris, JonathanWang, Jeh-JengGammone, M.A.Morris, David L.Wang, PengGanesan, AMorrissey, John P.Wang, TaoGarcia Camacho, F.Motterlini, RobertoWang, XuGarcía Nieto, Paulino JoséMourtas, SpyrosWang, San-LangGarcía-Caballero, MelissaMoyano, AlbertWaring, PaulGarneau-Tsodikova, SylvieMull, JeremyWeesepoel, YannickGarrett, TeresaMüller, Werner E.G.Wei, MeiGatti, LauraMulloy, BarbaraWei, Yu-HongGea, Oliveri ContiMundt, SabineWhalen, Kristen E.Genilloud, OlgaMuramoto, KojiWhite, Robert L.Gerssen, ArjenMurch, SusanWhite, AlexGervay-Hague, JacquelynMurphree, S. ShaunWichard, ThomasGhori, Muhammad UsmanMurphy, CarolineWielgosz-Collin, GaetaneGiddings, Lesley-AnnMurphy, Patrick J.Williams, BeckyGil, José A.Musgrave, IanWilliams, CraigGill, Chris I.R.Nagai, TakeshiWilliams, Gwyn T.Giussani, PaolaNagata, EiichiroWoo, Hee ChulGlaser, KeithNaito, YujiWormald, Mark R.Glibert, PatNaka, TetsujiWorman, JamesGoiris, KoenNaka, HiroakiWu, Yu-JenGoldberg, Inna KhozinNakajima, KatsuyukiWu, Chang-JerGolokhvast, KirillNam, Sang-JipWu, Yang-ChangGomes, Nelson G.M.Napier, JohnathanWu, Shih-HsiungGómez, XiomarNasopoulou, ConstantinaWu, Chi-ReiGomez-Matallanas, DavidNaughton, ViolettaWu, Tung-KungGonçalves, OlivierNeau, Steven H.Wu, HaoGonzález, Miguel A.Neilan, BrettWu, JianGonzález-Rodríguez, María-LuisaNestor, GustavXu, ChunlinGoswami, Rajib KumarNeugebauer, WitoldYamada, TakeshiGribble, GordonNewman, DavidYamada, ShuheiGribble, Gordon W.Newman, Mari-AnneYamaguchi, YoshikiGrogan, GideonNieden, Nicole I ZurYamaguchi, TakumiGrüschow, SabineNishimura, ShinichiYamashita, EijiGudmundsdottir, Bjarnheidur K.Ng, Tzi B.Yamashita, Mari YotsuGuella, GrazianoNoh, Jeong SookYan, NingGugliandolo, ConcettaNomikos, TzortzisYan, AixinGuihéneuf, FreddyNorambuena, FernandoYang, HongshunGulder, Tobias A. M.Northen, TrentYang, JyisyGunawardena, ShermaliNovakovic, KatarinaYarotskyy, ViktorGunia-Krzyżak, AgnieszkaNovelli, AntonelloYasui, HiroyukiGupta, PrasoonNowak-Sliwinska, PatrycjaYates, Edwin A.Guschina, Irina A.Nyman, Tuula A.Yatsunami, RieGustafson, KirkO’Doherty, GeorgeYonekura, MasamiGutierrez, TonyO’Gara, FergalYoo, Eun-SookHachiya, AkiraObata, ToshihiroYoshida, MasahiroHaertlé, ThomasOberlies, Nicholas H.Yoshida, HiroshiHalavaty, AndreiOchi, KozoYu, MingfengHamann, Mark T.Oda, TatsuyaYu, RongminHamilton, GerhardOddo, AlbertoZaharof, DavidHammond, Rosemarie W.Oh, Dong-ChanZan, JindongHampel, DanielaOhta, ShinjiZang, LiqingHansen, Pernille B.Oishi, NaokiZarros, ApostolosHarada, Ken-IchiOja, TerhiZhang, DongzeHardy, JoanOkamoto, YoshiharuZhang, FumingHarrison, Paul H. M.Oku, NaoyaZhang, HaoHartshorn, KevanOlenyuk, Bogdan Z.Zhang, GuojianHartung, JensOlinga, PeterZhang, FanHatakeyama, TomomitsuOliveira, P.a.Zhang, JieHatano, TsutomuOren, AharonZhang, ChangshengHayashi, YoshihikoOrjala, JimmyZhao, MingHelbert, WilliamOtsuka, HideakiZhou, AiminHentschel, UtePaerl, Ryan W.Zhu, LinHeras, AngelesPaglia, GiuseppeZhu, LiandongHerfindal, LarsPalmer, MikeZhukova, NataliaHernández-Becerril, David U.Pan, Cheol-HoZimmermann, KatharinaHess, PhillipPan, Bonnie SunZubía, EvaHessel, StefaniePang, Myung-GeolZvyagintseva, TatyanaHeyland, AndreasPant, Harish C.Zykova, TatyanaHidari, KazuyaParé, Paul W.


